# Longitudinal study of multiple sclerosis lesions using ultra-high field (7T) multiparametric MR imaging

**DOI:** 10.1371/journal.pone.0202918

**Published:** 2018-09-13

**Authors:** Sanjeev Chawla, Ilya Kister, Tim Sinnecker, Jens Wuerfel, Jean-Christophe Brisset, Friedemann Paul, Yulin Ge

**Affiliations:** 1 Center for Advanced Imaging Innovation and Research (CAI2R) and Bernard and Irene Schwartz Center for Biomedical Imaging, Departments of Radiology, New York, New York, United States of America; 2 Department of Radiology, Perelman School of Medicine at the University of Pennsylvania, Philadelphia, Pennsylvania, United States of America; 3 Department of Neurology, New York University School of Medicine, New York, NY United States of America; 4 Medical Image Analysis Center AG, Basel, Switzerland; 5 NeuroCure Clinical Research Center, Charité—Universitätsmedizin Berlin, corporate member of Freie Universität Berlin, Humboldt-Universität zu Berlin, and Berlin Institute of Health, Berlin, Germany; 6 Experimental and Clinical Research Center, Max Delbrueck Center for Molecular Medicine and Charité—Universitätsmedizin Berlin, Germany; Henry Ford Health System, UNITED STATES

## Abstract

Pathophysiology of multiple sclerosis (MS) lesions is dynamic and changes over time. The purpose of this exploratory study was to determine the longitudinal changes in MS lesions over time on ultra-high field MR imaging. Nine patients with MS underwent high-resolution 3D-susceptibility weighted imaging (SWI) and 2D-gradient-echo-T2*-weighted imaging on 7T MRI at baseline and after ~2.4 years of follow-up. Morphologic imaging characteristics, signal intensity patterns and quantitative susceptibility mapping (QSM) values of lesions were recorded at both time points. Lesions were classified as "iron-laden" if they demonstrated hypointense signal on T2*-weighted images and/or SWI as well as hyperintense signal on QSM. Lesions were considered "non-iron-laden" if they were hyperintense on T2*/SWI and isointense or hyperintense on QSM. Total of 162 non-iron-laden and 29 iron-laden lesions were observed at baseline. No change in baseline lesion size during follow up was recorded in 92.7%; no change in lesion-vessel relationship in 86.5%; and no change in signal intensity pattern in 96.9% of lesions. Three lesions which were non-iron-laden at baseline, exhibited iron at follow-up. In two iron-laden lesions, redistribution of iron content was observed at follow-up. Two-thirds of these iron-laden lesions showed an increase in QSM at follow-up relative to baseline, and the remaining one-third exhibited decrease in QSM. Most of the newly formed lesions (11/13, 84.6%) at follow-up were iron-laden. 7T multiparametric MRI is a useful tool for tracking the evolution of MS lesions, especially with regard to changes in iron content.

## Introduction

Multiple sclerosis (MS), a chronic autoimmune disease, causes lesions in the central nervous system that are characterized by variable degree of inflammation, demyelination, axonal injury, and iron accumulation [1]. MR imaging is routinely used to support the clinical diagnosis of MS, characterize disease activity, and monitor response to therapy. In a recent cross-sectional study, four distinct patterns of MS lesions were described based on signal intensity pattern on ultrahigh field (7T) gradient echo-T2* (GRE-T2*), susceptibility weighted imaging (SWI) and quantitative susceptibility mapping (QSM) [2]. This study also demonstrated that all lesions could be defined by MRI changes consistent with demyelination and inflammation, but that only a small number of lesions had evidence of iron accumulation either in nodular or ring-like pattern. These findings are consistent with previous histochemical studies [3, 4] that have shown that iron deposits were present only in a small subset of MS lesions. The molecular pathways for iron accumulation in MS lesions have been proposed to involve iron sequestered microglia or macrophages as well as iron accumulation in astroglia [5]. Because the cellular activity of MS lesions is dynamic, [6] the presence and spatial distribution of iron in MS lesions may vary as lesions evolve. Advancing our understanding of the longitudinal pathophysiological changes of MS lesions may provide new opportunities to monitor disease progression and to suggest novel therapeutic interventions.

A recent longitudinal study [7] with a mean follow-up of 3.5 years observed slow expansion of hypointense, iron-containing rim on SWI indicating that iron-laden lesions, in contrast to non-iron-laden lesions, may be actively evolving. However, another study [8] failed to detect morphological variations in either nodular or ring lesions during a 2.5-year follow-up using 7T GRE-T2* sequence derived phase images. The difference between studies may be due to relative insensitivity of phase images to reliably detect changes in tissue microstructures [9] and to quantify tissue iron content [10]. More advanced techniques, such as QSM, improve the detection and spatial distribution of subtle iron deposition that are not seen on conventional T2* imaging [11]. Longitudinal studies that used QSM have also reported temporal variations in lesion susceptibility as they evolve from contrast enhancing to non-contrast enhancing stage [12]. Thus, QSM appears to be a more promising tool to track the evolution of MS lesions than GRE-T2*.

The present study was performed to elucidate the longitudinal evolution of MS lesions with respect to size, iron content, lesion-to-venule relationship and lesion pattern using multiparametric 7T MR imaging that included GRE-T2*, SWI and QSM and to document emergence of new lesions.

## Materials and methods

### Subjects

This study was approved by the Institutional Review Board of New York University Langone Medical Center (NYULMC). A written informed consent was obtained from each patient. A cohort of nine MS patients (mean age: 54.1±13.3years; range = 36.0–70.3 years at baseline; 3 males/6 females) diagnosed with definite MS according to the revised MacDonald criteria [13] were recruited. The mean disease duration at enrollment was 15.7±12.4 years. The disease subtype was relapsing-remitting in 4 and progressive in 5 patients. At the time of the initial MRI, 5 patients could walk unassisted, 3 used a cane, and 1 needed a scooter. All patients underwent MR imaging at baseline and at a follow-up period. The time interval between the two MR imaging sessions ranged from 2.00 to 3.83 years (mean = 2.40±0.56 years). The demographic and disease-related information on each patient is summarized in **Table 1**.

**Table 1:** Demographics of MS patients.

ID	Age at Baseline (Years)	M/F	Race	Disease Type	Disease Duration (Months)	EDSS	DMT	Time between baseline and Follow up MRI (Years)	Number of Lesions at Baseline	Number of Lesions at Follow-up
1	67.7	F	AA	PPMS	11	6	Cyclophosph-amide	2.43	67	67
2	48.9	M	AA	SPMS	14	2	No DMT	2.46	15	25
3	72.8	F	AA	SPMS	15	2	Glatiramer- acetate	2.03	13	13
4	48.0	F	His	RRMS	7	1	No DMT	2.06	19	19
5	36.0	M	CAU	RRMS	13	4	Dimethyl fumarate	2.26	2	2
6	40.8	F	CAU	RRMS	12	1	Fingolimod	2.00	21	21
7	70.4	F	CAU	SPMS	48	7	Natalizumab	2.39	17	17
8	55.9	F	AA	SPMS	12	6	Dimethyl fumarate	3.83	16	17
9	48.0	F	CAU	RRMS	9	1	Rituximab	2.09	21	23

M = Male; F = Female; AA = African-American; His = Hispanic; CAU = Caucasian; PPMS = Primary Progressive MS; SPMS = Secondary Progressive MS; RRMS = Relapsing-Remitting MS; EDSS = Expanded Disability Status Scale; DMT = Disease Modifying Therapy

### Ultra-high field MRI and data post-processing

All patients underwent ultra-high field MR imaging using whole-body 7T human MR systems (MAGNETOM, Siemens Medical Solution, Erlangen, Germany) equipped with 24-channel phased array coil (Nova. Medical, Inc., MA). The imaging protocol included a high-resolution axial 2D-GRE-T2* weighted imaging, high-resolution axial 3D-SWI, T2-FLAIR, and sagittal T1-weighted 3D-MPRAGE sequences. To avoid major susceptibility artifacts from air-tissue interfaces, only supratentorial brain regions were covered while acquiring 2D-GRE-T2* and 3D-SWI. None of the patients received intravenous contrast agent. The acquisition parameters for GRE-T2*weighted imaging were: TR/TE = 580/25ms, flip angle = 35°, slice thickness = 2 mm, FOV = 240×240mm^2^, voxel size = 0.2×0.2mm^2^, for sagittal 3D-SPACE-FLAIR, TR/TE/TI = 8000/380/2100ms; isotropic voxel size = 1.0×1.0×1.0mm^3^ and for sagittal T1-weighted 3D-MPRAGE, TR/TE/TI = 2000/2.92/1100ms, isotropic voxel size = 1.0×1.0×1.0 mm^3^

High-resolution, flow-compensated 3D-SWI images were acquired with the following parameters: TR/TE = 27/18ms, flip angle = 18°, slice thickness = 2mm, FOV = 240×240mm^2^, base resolution = 1024, voxel size = 0.23×0.23×2mm^3^, bandwidth = 110Hz/px, acquisition time = 7:49min, and iPAT factor = 2.

The source magnitude and phase images from each SWI scan were obtained and were used to generate SWI venograms. All phase images were reconstructed and corrected for field inhomogeneities with Hanning high-pass filter of size 96×96 using SPIN image processing software (signal processing in MR http://www.mrc.wayne.edu). The original magnitude image was multiplied by the phase mask four times in order to enhance the visibility of lesions and venous structures. Finally, SWI venograms were created by performing minimum intensity projection (mIP) over 2 contiguous slices (4mm thick).

Susceptibility weighted imaging and mapping (SWIM) algorithm developed by Haacke’s group [14, 15] was used to reconstruct QSM maps from high resolution 3D-SWI data. The post-processing involved skull stripping to remove the artifacts caused by skull and brain tissue interface using the brain extraction tool, followed by phase unwrapping using a Laplacian operator. To remove background field inhomogeneity, a variable high-pass filter of 32 pixels size was applied and, finally, inverse filtering was performed to generate QSM maps.

### Data analysis

All MR images (GRE-T2*, SWI and QSM) were analyzed and interpreted at both time points by two investigators jointly. These investigators worked together in achieving the final consensus. In event of disagreement in evaluating data–which occurred with regard to 2 lesions—a senior author with over 20 years of experience in the field of advanced MR imaging in MS was asked to provide guidance.

The lesion count and location of MS lesions were evaluated. Additionally, the following morphological imaging characteristics were recorded for each lesion: 1) largest cross-sectional diameter, 2) presence of one or multiple central intralesional venules; 3) differential signal intensity within the lesions and 4) presence of a peripheral iron rim. Based upon the largest cross-sectional diameter, lesions were classified as small (<2mm), medium (2-5mm) and large (>5mm). We classified MS lesions into four distinct patterns based on lesion signals on GRE-T2*, SWI and QSM; putative correlation of each pattern with underlying pathology are summarized in **Table 2**. Pattern A lesions were hyperintense on GRE-T2* weighted images, hyperintense or isointense on SWI, and isointense (inconspicuous) on QSM. Pattern B lesions were hyperintense on GRE-T2* and hyperintense on QSM. Pattern A and B lesions were considered as ‘non-iron enriched’ lesions, while lesions which demonstrated hypointensity on GRE-T2* and/or SWI and hyperintensity on QSM, were considered ‘iron-laden’. The iron-laden lesions were classified as ‘nodular’ (Pattern C) or ‘peripheral rim’ (Pattern D). To evaluate quantitative temporal variations in iron content, QSM values for pattern C and D lesions were computed. Additionally, QSM values were computed for the ‘newly formed’ iron-laden lesions (those present only on follow-up MRI, but not on baseline MRI).

**Table 2:** Proposed histopathologic interpretation based upon signal intensity patterns on MR images.

Tissue Content	Susceptibility Effect	Signal Intensity on GRE-T2*	Signal Intensity on SWI	Signal Intensity on QSM
Calcium	Diamagnetism	Hypointense	Hypointense	Hypointense
Myelin	Diamagnetism	Isointense	Isointense	Isointense
Variable degree of micronecrosis, edema, gliosis, demyelination and macromolecules	Diamagnetism	Hyperintense	Isointense or Hyperintense	Isointense
Extensive degree of demyelination	Loss of diamagnetism (paramagnetism)	Hyperintense	Isointense or Hyperintense	Hyperintense
Iron	Paramagnetism	Hypointense	Hypointense	Hyperintense

GRE = Gradient Echo; SWI = Susceptibility Weighted Imaging; QSM = Quantitative Susceptibility Mapping

QSM values were computed from lesions by manually drawing ROIs. Since the use of high pass filter while reconstructing QSM maps may reduce the calculated iron content from different tissue compartments, we believe this process might have resulted in underestimation of the QSM values computed from iron and non-iron-laden lesions. In order to correct for the QSM values, a simulation algorithm [15] was used to obtain a scaling factor based upon the size of the lesions. This size dependent scaling factor was multiplied by the original QSM values to obtain corrected QSM values for each lesion.

### Statistical analysis

Kolmogorov-Smirnov tests were used to determine the nature of QSM data distribution. As the data showed departure from Gaussian distribution, non-parametric Mann-Whitney-U tests were performed to evaluate the differences in QSM between two time points. A probabilistic (p) value of less than 0.05 was considered significant. All statistical analyses were performed by using a statistical package, SPSS for Windows (version 18.0; SPSS Inc., Chicago, III, USA)

## Results

### Lesion count, size and location

A total of 191 lesions (mean = 21.2/patient, range 2–67) were observed at baseline, and a total of 204 lesions were observed at follow-up (mean = 22.7/patient, range: 2–67), a 6.8% increase in lesion count during the follow-up (**Table 1**). The 13 new lesions were observed in 3 out of 9 patients. At baseline, majority of the lesions (n = 117, 61.2%) were small, and minority were medium (n = 58, 30.4%) or large (n = 16, 8.4%). At follow-up, distribution of lesions by size was similar to that at baseline: small (n = 123, 60.3%), medium (n = 63, 30.9%) and large (n = 18, 8.8%). A great majority of baseline lesions (n = 177/191; 92.7%) did not undergo any appreciable change in size at follow-up. However, a total of 10 lesions that were small at baseline were of medium size at follow-up, while 4 lesions that were medium at baseline, were classified as small at follow-up.

Most of the baseline lesions were located in subcortical white matter (75.9%), followed by periventricular white matter (14.1%), juxta-cortical white matter (5.3%) and corpus callosum (4.7%). Majority of new lesions (8 out of 13, 61.5%) were also located in subcortical white matter region.

### Lesion pattern

**Fig 1:**
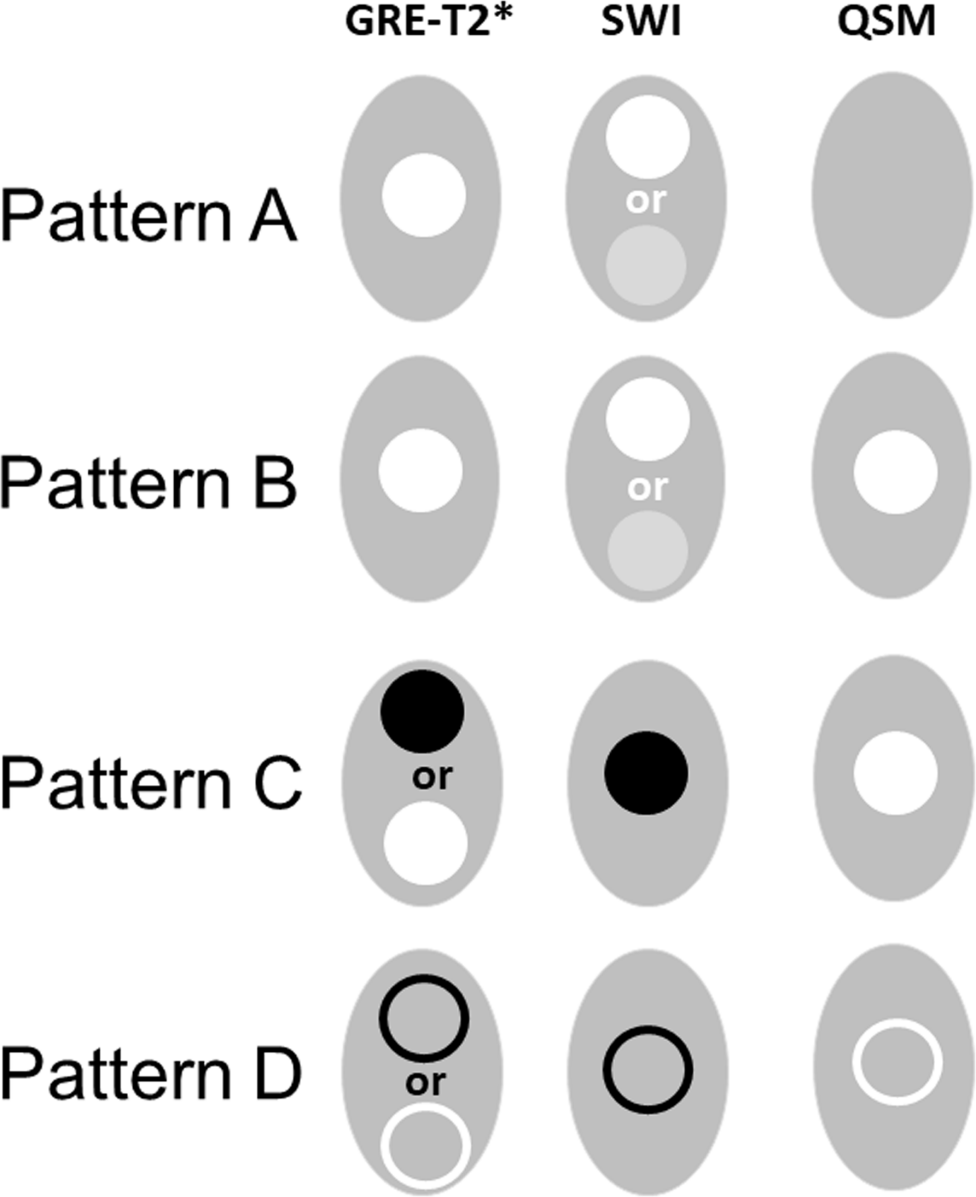
Schematic sketches for lesions depicting ideal signal intensity distribution on images. The ideal signal intensity distribution on GRE-T2* weighted image, SWI and QSM for Pattern A to D lesions are shown. Pattern A and B lesions belong to non-iron related pathology, whereas Pattern C (nodular) and D (ring-like) lesions are associated with iron-enriched pathology.

**Fig 2:**
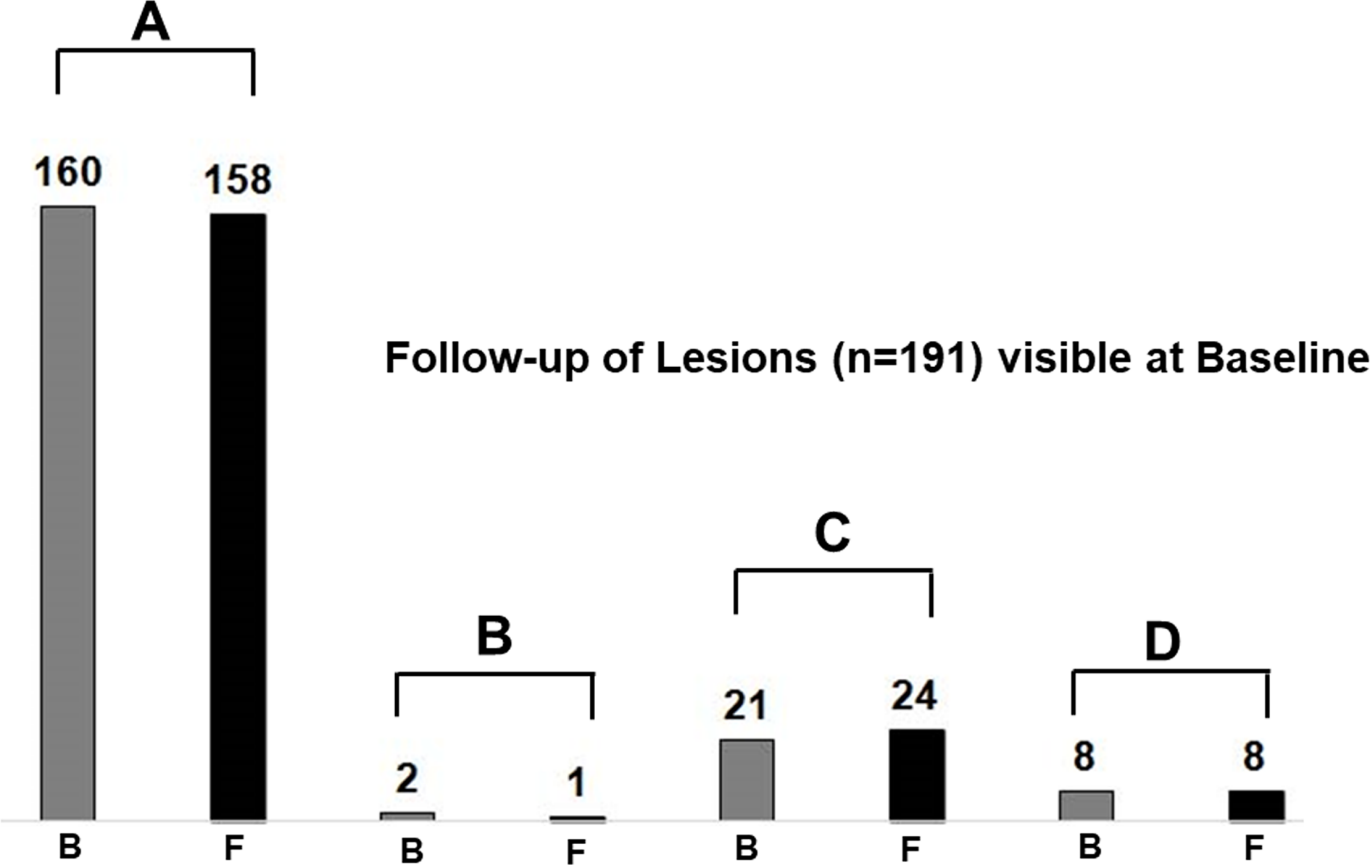
Distribution of total number of lesions at baseline and at follow-up period. Bar diagram showing the variations in the number of baseline lesions (n = 191) of each pattern (A-D) between baseline (gray-color) and follow-up period (black-color).

**Fig 3:**
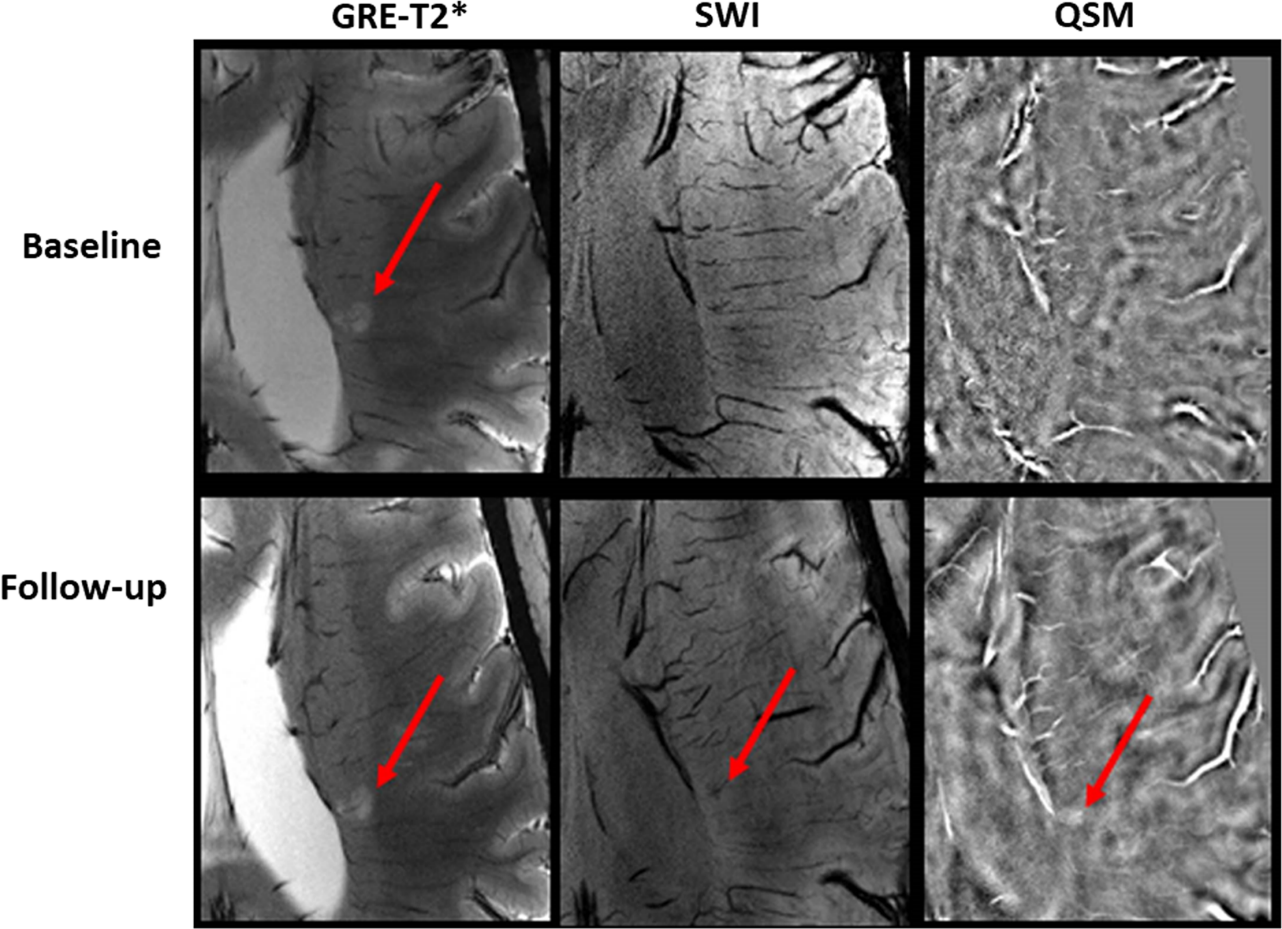
Pattern switch from non-iron related pathology at baseline to iron related pathology at follow-up. In the upper rows, axial GRE-T2* weighted image shows a hyperintense lesion (arrow) that appears isointense on SWI and QSM consistent with Pattern A at baseline. This lesion exhibited hypointensity on SWI and hyperintensity on QSM (lower rows) at follow-up indicating iron accumulation. Hence this lesion switched from Pattern A at baseline to Pattern C after 2 years of follow up.

**Fig 4:**
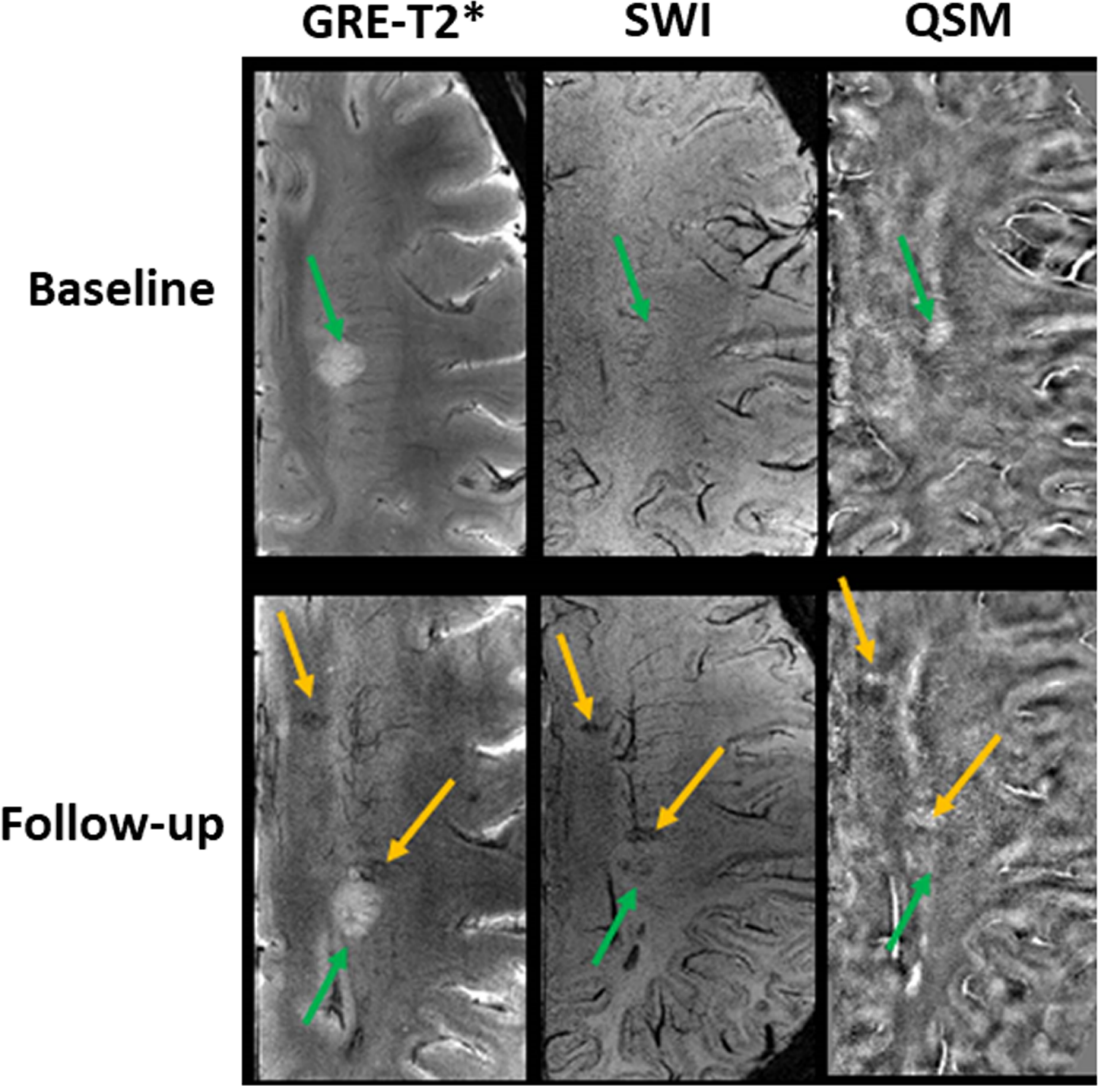
No change in pattern B lesion and appearance of new iron-laden lesions at follow-up. In the upper rows, axial GRE-T2* weighted image shows a hyperintense lesion (green arrow) that appears isointense on SWI and hyperintense on QSM consistent with Pattern B at baseline. This lesion showed similar signal intensity pattern on images (lower rows) at follow-up suggesting this lesion remained as Pattern B at follow-up during a period of 2.5 years. Additionally, two lesions that were not visible at baseline, became visible at follow-up. Incidentally, both of these lesions demonstrated nodular hypointense signals on GRE-T2* and SWI (yellow arrows) and hyperintense signal on QSM (yellow arrows) consistent with Pattern C.

As described previously [2], four morphologically distinct patterns of lesions could be distinguished in the present study (**Fig 1**). The distributions of these four-distinct pattern of lesions at baseline (n = 191) and at follow-up are shown in **Fig 2**. Vast majority of lesions (96.9%) showed no appreciable change in pattern during follow up, i.e. there was no change in the signal intensity distribution of these lesions on GRE-T2*, SWI and QSM at the two time-points. Pattern switches were observed in only 6 lesions (3.1% of total initial lesion count) in 4 patients. Of 191 lesions that were visible at baseline, most of the lesions (n = 160) had Pattern A that persisted in 157/160 (98.1%) of lesions at follow-up. Three ‘Pattern A’ lesions at baseline, switched to Pattern C on follow-up **(Fig 3)**. There was no change in the signal intensity distribution in one Pattern B lesion between two-time points **(Fig 4)**, while another baseline Pattern B lesion switched to Pattern A at follow-up. Thus, the total number of lesions with Pattern A at follow-up was 158 (**Fig 2**).

**Fig 5:**
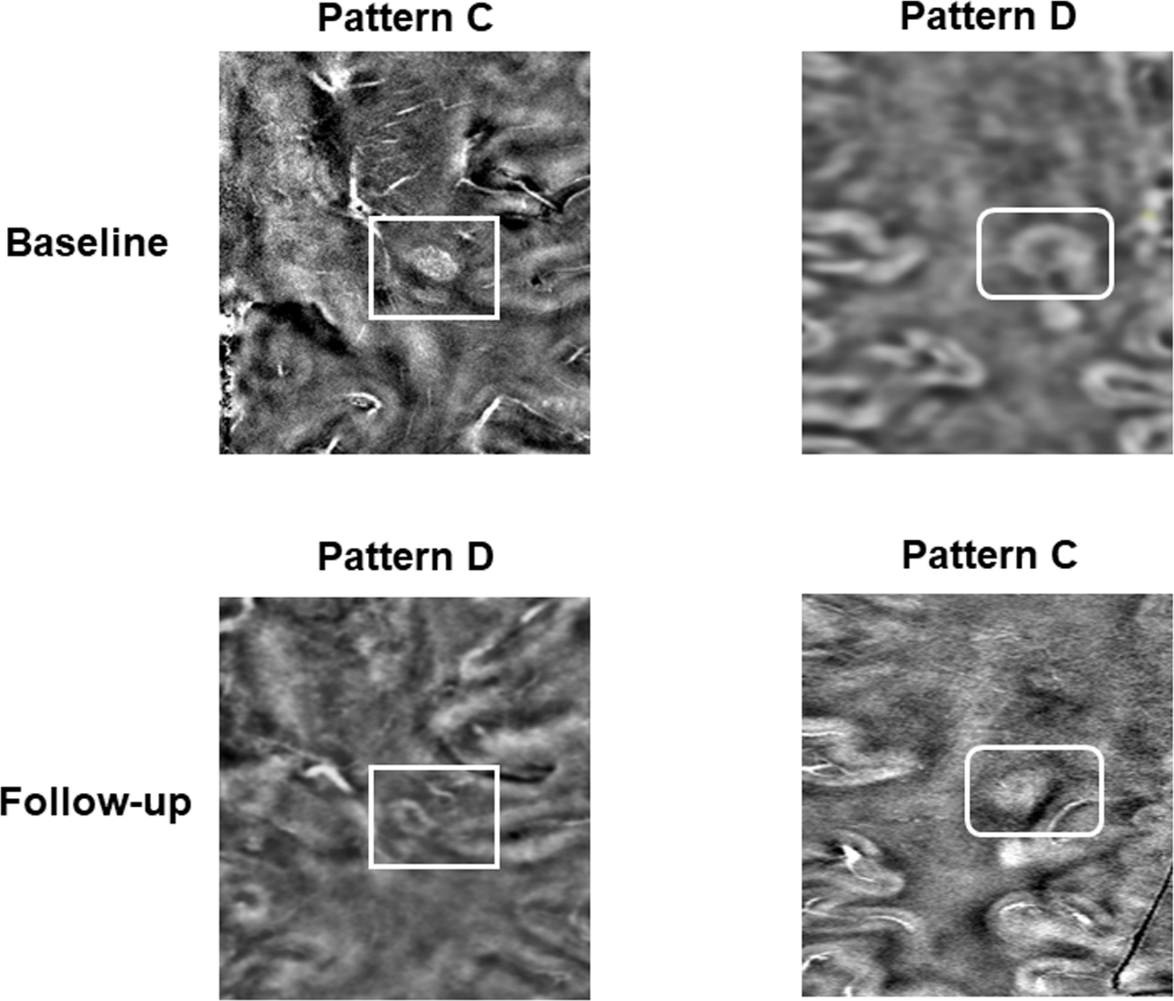
Redistribution of signal intensity pattern in iron-laden lesions at follow-up relative to baseline. In the upper left panel, QSM shows solid hyperintense signal within a lesion indicating iron-laden nodular lesion (Pattern C) at baseline. This lesion underwent redistribution of signal intensity from nodular to ring-like (Pattern D) on QSM (lower left panel) at follow-up. In the upper right panel, QSM shows ring-like hyperintense signal within another lesion consistent with Pattern D at baseline that underwent redistribution of signal intensity from ring-like to nodular (Pattern C) on QSM (lower right panel) at follow-up.

All the 29 iron-laden lesions [Pattern C (nodular, n = 21) and Pattern D (ring-like, n = 8)] observed at baseline retained iron content at follow-up. Because of pattern switch of three baseline Pattern A lesions to Pattern C at follow-up, there was a small increase in the number of Pattern C lesions (from 21 to 24) at follow-up. In two iron-laden lesions, there was redistribution of signal intensity (from nodular to ring-like in one lesion and from ring-like to nodular in another lesion) **(Fig 5)**; thus, total count of Pattern D lesions remained same (n = 8) at follow-up.

### Appearance of new lesions on follow-up MRI

A total of 13 new lesions were observed at follow-up in three patients. Majority of these new lesions (n = 11/13; 84.6%) were iron-enriched **(Fig 4)**. Of 11 new iron-laden lesions, 9 were nodular and two had ring-like pattern. Additionally, two new Pattern A lesions appeared at follow-up.

### Lesion-vessel relationship

Central venule was identified in 133 (70%) of lesions at baseline. The proportion of lesions with central venules by lesion patterns was as follows: Pattern A (n = 110/160, 68.8%); Pattern B (n = 2/2, 100%); Pattern C (n = 13/21, 62%); and Pattern D (n = 8/8, 100%). There was no significant difference (p>0.05) in the presence of central venule between non-iron-laden (n = 112/162, 69.1%) and iron-laden lesions (n = 21/29, 72.4%). On follow up, central venule within a lesion was seen in 123 out of 133 lesions in which a central venule was seen at baseline (93%). Ten lesions with a venule at baseline did not display a venule on follow up. On the other hand, in 8 lesions in which venule could not be seen at baseline, a venule was detected at follow up. Of the new lesions that were seen only on follow up, 8 out of 13 (61.5%) lesions had a central venule.

### Variation in QSM values

**Fig 6:**
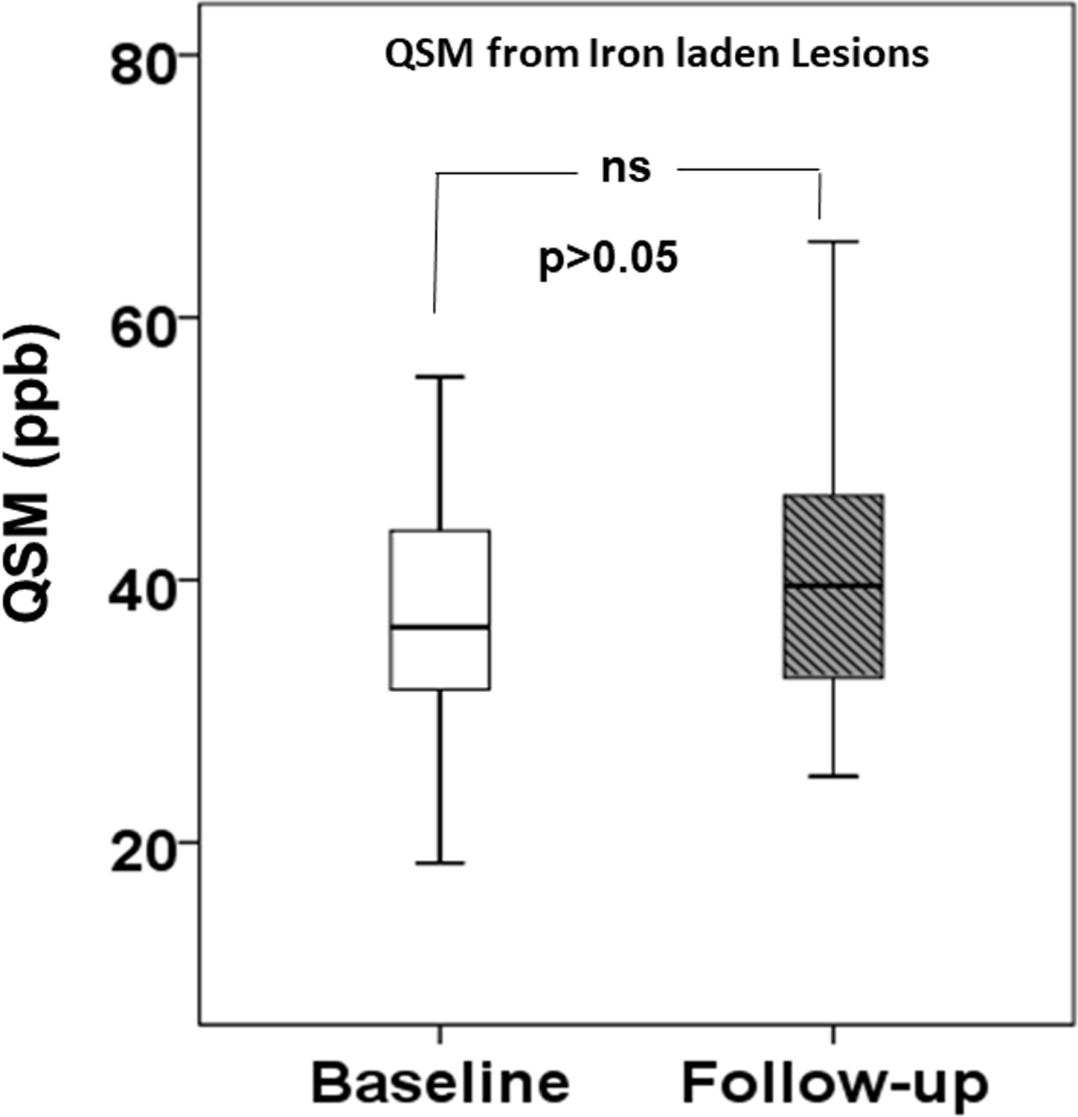
Distribution of QSM from iron-laden lesions at baseline and at follow-up period. Box-and-whisker plots demonstrating the distribution of QSM from iron-laden (Pattern C and Pattern D) lesions at baseline (*white*) and at follow-up (*shaded*). The bottom and top edges of boxes represent the 25^th^ percentile, and the 75^th^ percentile values. The bands within the boxes represents 50^th^ percentile (median). Whiskers display the range of data distribution. Non-significant (p>0.05) increase in QSM at follow-up relative to baseline was observed from these lesions.

By definition, all Pattern A lesions demonstrated isointense signal intensity on QSM, i.e. they were inconspicuous on these maps. One Pattern B lesion did not undergo any pattern switch yet showed a decrease in the QSM from baseline (30.27ppb) to follow-up (14.02ppb). Iron-laden Pattern C and Pattern D lesions demonstrated non-significant (p>0.05) increase in mean QSM from baseline (35.7±13.4ppb) to follow-up (38.1±13.2ppb, **Fig 6**). Most of iron-laden lesions (n = 19) showed an elevation in QSM at follow-up relative to baseline. However, some lesions (n = 10) exhibited decrease in the QSM values. The mean QSM for newly formed iron-laden lesions (n = 11) was 41.8±16.1ppb.

### Distribution of lesions by disease type

Iron-laden lesions (Pattern C and D) were present in 5/9 (55.5%) patients at baseline. Interestingly, 4 of these 5 patients (80%) were in the progressive stage of the disease [PPMS (n = 1) and SPMS (n = 3)]. Of 29 iron-laden lesions, 27 (93.1%) were present in the 4 patients with progressive disease, and only 2 (6.9%) lesions were present in the 1 patient with RRMS. Out of 13 newly formed lesions, 11 developed in 2 patients with progressive disease and other 2 lesions in a patient with RRMS. The details on existing iron-laden and non-iron-laden lesions at baseline and at follow up, as well as documentation of new lesions by disease type are shown in **Table 3**.

**Table 3:** Distribution of iron-laden and non-iron-laden lesions at baseline, pattern switch and formation of new lesions by disease type.

**Distribution of Iron-laden and Non-iron-laden Lesions (n = 191) at Baseline**				
Patient ID	Disease Type	Number of Iron-laden Lesions	Number of Non Iron-laden Lesions	Total Number of Lesions
1	PPMS	1	66	67
2	SPMS	12	3	15
3	SPMS	0	13	13
4	RRMS	0	19	19
5	RRMS	0	2	2
6	RRMS	0	21	21
7	SPMS	1	16	17
8	SPMS	13	3	16
9	RRMS	2	19	21
**Pattern Switch (n = 6)**				
Patient ID	Disease Type	Lesion Pattern at Baseline	Lesion Pattern at Follow-up	
2	SPMS	A	C	
2	SPMS	A	C	
2	SPMS	C	D	
4	RRMS	B	A	
6	RRMS	A	C	
8	SPMS	D	C	
**Formation of New Lesions (n = 13) at Follow-up**				
Patient ID	Disease Type	Iron-laden Lesion	Non-iron-laden Lesion	
2	SPMS	10	0	
8	SPMS	1	0	
9	RRMS	0	2	

PPMS = Primary Progressive MS; SPMS = Secondary Progressive MS; RRMS = Relapsing-Remitting MS

## Discussion

In this exploratory longitudinal multiparametric ultra-high field MRI study of nine MS patients with a mean age of 54 years, we observed a 7% increase in total lesion count during a follow up period of 2.4 years. This relatively small increase may be due to the use of disease-modifying therapies that effectively prevent formation of new lesions in 5/7 of our patients [16, 17] and the relatively older age of cohort, when new lesion formation is less common [18]. A vast majority of these lesions did not demonstrate appreciable change in size, signal intensity pattern and lesion-vessel relationship during follow-up. Only 6 lesions (3%) demonstrated pattern switch at follow-up, mostly relating either to increase in iron content (3 lesions) or redistribution of iron within lesions (2 lesions). The vessel-lesion relationship remained unchanged in 181 (94.8%) lesions, while the remaining 10 lesions had an approximately equal chance of apparent ‘loss’ or ‘gain’ of venule on follow up that might be incidental or related to technical factors, such as slice angulation or partial volume effects.

Of 13 lesions seen at follow-up that were not present at baseline, 11 were iron-laden (Pattern C or D). This finding would be in line with the notion that rapid iron accumulation is a hallmark of the active MS lesion formation, while the iron-containing inactive lesions generally show only slow change in iron content [19]. The molecular pathways for iron accumulation in MS lesions remain to be fully understood. Several plausible biological mechanisms include deposition of iron-rich oligodendrocyte debris, iron sequestered activated microglia or macrophages, product of local microhemorrhages following venule wall damage and hypoxia-induced over-expression of iron-regulating proteins and subsequently elevated translation of transferring receptors [4, 6, 20], and accumulation of iron in reactive astroglia in the form of ferritin.

At baseline, a great majority of the lesions (n = 160/191, 83.8%) demonstrated hyperintense signal on GRE-T2* and /or SWI and isointense signal on QSM indicating no or minimal iron deposition (Pattern A). Nearly all Pattern A lesions (157/160) showed no change in the signal intensity pattern over follow-up period. These findings suggest that these lesions were in the chronic inactive stage characterized by diminished inflammatory activity and varying degree of neuronal/axonal loss, micronecrosis, gliosis, and no significant changes in the number of iron-containing macrophages/microglia and lymphocytes [21]. Presumably, these are the same lesions that have also been characterized by rare or absent iron-containing oligodendrocytes on histochemistry [19]. However, three Pattern A lesions accumulated iron content in the nodular form and were therefore re-classified as Pattern C lesions at follow-up. One could explain this pattern switch by invoking the existence of two distinct phenotypes of macrophages with different effector functions [22, 23]. One population comprises of classically activated macrophages with M1-polarization that are iron rich and are also known to be neurotoxic via secretion of high levels of proinflammatory cytokines and toxic intermediates. Alternatively activated or M2-polarized macrophages release intracellular iron and are essential for tissue repair as these cells possess efficient phagocytic capacity. Cells of the monocyte-macrophage lineage are characterized by considerable plasticity and can change their functional state and give rise to different cell populations in response to different environmental signals [24, 25]. It is possible that dynamic phenotype alteration of M2-polarized macrophages to M1-polarized population of cells might be responsible for pattern switch from non-iron to iron pathology in these three. Correlative MR imaging with histopathological and immunohistochemical studies are required to fully understand the pathophysiological mechanisms for pattern switching in MS.

In agreement with previous studies, [7, 26] only a small number of lesions were iron-laden (~15%) at baseline in our patients. Majority of these iron-laden lesions did not show any shift in the signal intensity pattern. This is in line with a prior observation that iron-laden MS lesions do not show obvious variation in morphology on phase images during a 2.5-year follow-up [8]. Iron-enriched lesion patterns could be due to accumulation of iron-laden activated macrophages [3, 4]. It is unclear why the macrophages could persist there for such a long time. A similar situation may be seen in intracerebral hemorrhage, where perivascular collections of iron in hemosiderin within macrophages may be visible indefinitely on MR imaging [27]. Another possible reason for persistence of iron pattern within lesions may be due to the presence of iron-enriched astrocytes. Since astrogliosis is considered to be a pathological hallmark of chronic lesions, heme-oxygenase-I enzyme may promote sequestration of iron in the oxidative stressed astrocytes. These iron-laden astrocytes can survive for prolonged periods due to their robust anti-oxidant defenses, cytoprotection, and greater capacity for anaerobic metabolism compared to other cells in the brain [28].

Intriguingly, two of iron-laden lesions underwent reorganization of iron content—one from nodular to ring-like and another from ring-like to nodular in the present study. We believe that we were able to observe these transitions in iron patterns because high resolution (in-plane resolution:230 x 230μ^2^) SWI and QSM images were used in the current study. Additionally, QSM are known to more accurately resolve the magnetic susceptibility spatial pattern compared to phase images and hence depict both solid and rim patterns of susceptibility more precisely and reliably [11]. QSM may be useful to more accurately distinguish lesion's magnetic susceptibility distribution signature. A ring-like pattern may indicate the presence of iron-containing macrophages or astrocytes at the rim of a chronic active lesion whereas nodular pattern may represent the uniform distribution of cellular or extracellular sources of iron [19].

In addition to more accurately resolving underlying susceptibility pattern, QSM enables the quantitative assessment of iron accumulation and degree of demyelination within a tissue. Prior studies [2, 4, 29, 30] have reported QSM values in MS lesions on 7T MRI. Collectively, these studies have suggested that susceptibility MR imaging provides clinically relevant characterization of MS lesions. In the present study, a minority of lesions (34.5%) exhibited a decline in QSM values but most of the lesions (65.5%) demonstrated an increase in QSM at follow-up. On the whole, mean QSM across all the lesions did not change significantly during follow up. Using QSM, prior studies [12, 31] have shown that lesion susceptibility values increase sharply as enhancing lesions turn to non-enhancing lesions. Moreover, these susceptibility values remain high for several years (~4-years) followed by release of iron into the extracellular space concomitantly with switching of iron-laden chronic active lesions into non-iron containing chronic inactive silent lesions [31]. Our data suggests a trend toward higher QSM values over relatively short follow-up. It would be interesting to study QSM trajectories over a longer timescale. Taken together, these findings suggest that iron metabolism within the MS lesions is probably a slow but dynamic process correlating closely with the fluctuating inflammatory activities.

In our study, only 55.5% of the patients had iron-laden lesions and of these patients, 80% had progressive form of MS. Indeed, very few iron lesions were noted in patients with relapsing MS while 93%, of iron-laden lesions were present in patients with progressive disease. These findings indicate that advanced stage of disease is associated with abnormal iron metabolism. Indeed, elevated levels of ferritin have been observed in cerebrospinal fluid of patients with progressive disease [32]. However, we also observed that iron is frequently present in newly formed lesion–in fact, it was much more common in the new lesion than in existing lesions. Thus, one could hypothesize that iron accumulation occurs in newly formed lesions, followed by loss of iron in most but not all lesions.

Although we considered lesions that demonstrated hypointensity on GRE-T2* and/or SWI and hyperintensity on QSM as iron laden, other paramagnetic metals may provide similar signal intensity pattern. Another potential cause of hyperintense signal on QSM is extensive demyelination (loss of diamagnetism). However, we believe that hyperintense lesions on QSM observed in our study were most likely secondary to deposition of paramagnetic substances such as iron as demyelinated lesions would have been expected to show hyperintense signal intensity on GRE-T2* and/or SWI, which was not observed in our study.

Main limitation of our current study is its small sample which is due in part to the technical difficulties of using multiparametric technique and long follow-up required. Despite the small sample size, our study demonstrates the potential of multicontrast ultra-high field MR imaging in advancing our understanding of lesion evolution in MS and offers new and interesting findings. To obtain a more comprehensive understanding of the evolution of MS lesions, future studies would need to include a broader range of patients with different ages and MS subtypes. Our study was also limited by the fact that we used a relatively short follow-up duration of ~2.4 years. Another shortcoming of the current study was that R2* or T2* mapping was not performed due to time constraints; these sequences may provide additional quantitative information for characterization of MS lesions.

In conclusion, our findings indicate that combined analysis of ultra-high field (7T) GRE-T2*, SWI and QSM studies may help to track the evolution of MS lesions associated with both iron related and non-iron related pathologies. During the course of 2.4-year follow-up, majority of lesions did not show any obvious change in the signal intensity pattern, suggesting that most existing MS lesion plaques, once formed, tend to remain relatively inactive. A small number of lesions underwent pattern switches and most of the newly formed lesions were iron-enriched. Our findings provide preliminary insights into the pathophysiological features of MS lesions in particular the evolution patterns of iron deposition and lesion-vein relationship during the course of disease.

## Supporting information

S1 File This is the S1 Data File. (XLSX)
